# Differentiation between IgG4-related Mikulicz disease and Sjögren’s syndrome: A review case report and literature review

**DOI:** 10.1097/MD.0000000000032617

**Published:** 2022-12-30

**Authors:** Yurie Otani, Tomotaka Shimura, Taketoshi Nogaki, Yoichi Ikenoya, Koichiro Oyake, Naomi Imaizumi, Yukiko Inoue, Shuhei Uruma, Sawa Kamimura, Yojiro Kawamura, Sei Kobayashi

**Affiliations:** a Department of Otorhinolaryngology, Kanto Rosai Hospital, Nakahara-ku, Kawasaki, Japan; b Department of Otorhinolaryngology, Showa University Fujigaoka Hospital, Aoba-ku, Yokohama, Japan; c Department of Otorhinolaryngology, Showa University Koto Toyosu Hospital, Koto-ku, Tokyo, Japan.

**Keywords:** IgG4, IgG4-related disease, otologic manifestations, Mikulicz’s disease, Sjögren syndrome

## Abstract

IgG4-related diseases cause lesions in various organs throughout the body. In otorhinolaryngology, IgG4-related Mikulicz’s disease is suspected and diagnosed based on the presence of lesions of the head and neck, salivary and lacrimal gland enlargement, and bilateral sinus opacity concentrated on the maxillary sinuses. However, in some cases, it is necessary to consider about differentiation between IgG4-related Mikulicz’s disease and Sjögren syndrome.

**Patient concerns and diagnosis:**

A 75-years-old male patient visited our hospital with bilateral otitis media with effusion, which was resistant to conservative treatment. Other symptoms at presentation included enlarged bilateral submandibular and sublingual glands marked oral dryness, severe decrease in saliva secretion (1 mL/10 minutes), and dry eyes. We conducted a Schirmer’s and fluorescent dye tests, both of which were positive. High serum IgG4 levels were observed, and although the Sjögren syndrome (SS)-A/SS-B antibodies were negative, marked hypolacrimation and tear secretion were observed. Therefore, a detailed examination considering both IgG4-related Mikulicz’s disease and SS was conducted. Salivary gland scintigraphy performed prior to the salivary gland biopsy revealed a marked decrease in uptake, which satisfied the diagnostic criteria for SS; however, it was difficult to diagnose IgG4-related disease based on the diagnostic definition.

**Intervensions:**

Although a definitive diagnosis of SS was made, the persistent otitis media with effusion that was resistant to conservative treatment and bilateral mixed hearing loss were confirmed. As mixed hearing loss is considered an otological symptom of IgG4-related disease, oral steroid treatment was administered.

**Outcome:**

Thereafter, marked recovery of hearing and reduced swelling and induration of the bilateral parotid and submandibular glands were observed. Clinically, IgG4-related Mikulicz’s disease was strongly suspected, but a definite diagnosis of SS was made.

**lessons:**

In the absence of an IgG4-related Mikulicz’s disease diagnosis, careful differentiation between IgG4-related Mikulicz’s disease and 2 diseases and their diagnostic criteria was essential.

## 1. Introduction

IgG4-related disease (IgG4-RD) can cause lesions in various organs throughout the body. However, in the field of otorhinolaryngology, clinical findings in the head and neck, such as salivary and lacrimal gland enlargement, and sinus opacity may lead to suspicion of IgG4-RD and ultimately, a diagnosis. Although the disease is not common in the otolaryngology field, IgG4-RD should be kept in mind in daily clinical practice. Additionally, IgG4-related Mikulicz’s disease, which is particularly important in the field of otorhinolaryngology, often needs to be differentiated from Sjögren’s syndrome (SS) due to similarities in their respective clinical symptoms. Careful interpretation of the diagnostic criteria is important to differentiate IgG4-related Mikulicz’s disease and SS. In this report, we discuss our experience of a patient with severe otitis media with effusion (OME) and mixed hearing loss who responded well to oral steroid treatment at our institution. In this research, personal information was anonymized so that individuals could not be identified. This study has been approved by the research ethics committee of Showa University Fujigaoka Hospital (permission number: 22-176-B). Additionally, written informed consent was obtained from the patient and the patients’ guardian for publication of this case report and accompanying images.

## 2. Case presentation

**Figure 1.**
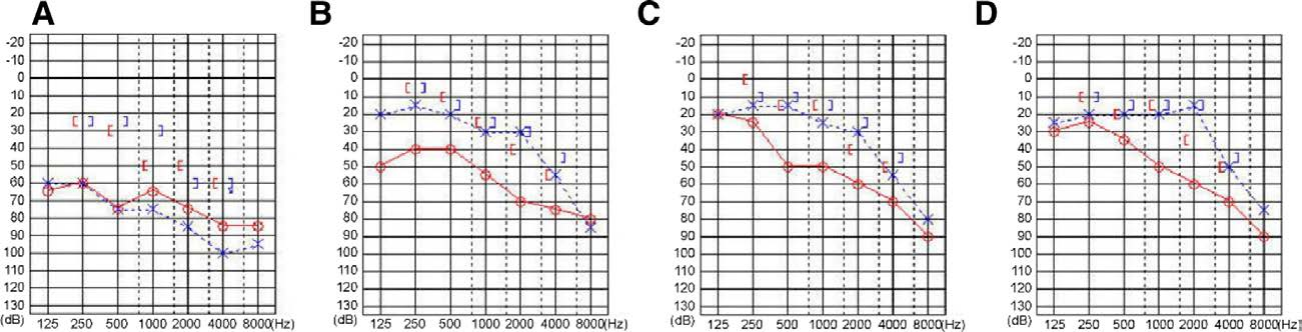
Changes in Pure Tone Audiometry Results. (a): At the first visit to our department, a relatively severe bilateral mixed hearing loss is observed. (b): Approximately 2 weeks after the start of oral steroid treatment, 30 mg/day of oral PSL is administered. Significant improvement in both air conduction and bone conduction hearing is observed on both sides. (c): Approximately 8 weeks after the start of oral steroid treatment, 25 mg/day of PSL is administered. Further improvement in both air conduction and bone conduction hearing is observed on both sides. (d): Approximately 20 weeks after the start of oral steroid treatment, 15 mg/day of PSL is administered. About 16 weeks after the start of treatment, when the dose is reduced to 10 mg/day of PSL, the patient’s hearing begins to deteriorate, so the dose is increased again to 15 mg/day, and the hearing improves. As such, this is considered the maintenance dose. PSL = prednisolone.

Our patient was a 75-years-old male who had received long-term treatment with low-dose macrolide for persistent ear fullness and OME at the department of otorhinolaryngology at another hospital. Treatment was unsuccessful. The patient visited our department with dry mouth symptoms, and had a history of bronchial asthma, which was controlled by the administration of inhaled corticosteroid/long-acting muscarinic antagonist/long-acting *β*2-agonist prescribed by the department of internal medicine at our hospital. Examinations conducted at our department revealed OME with bilateral tympanic membrane depression and a slight yellow middle ear effusion. Audiometry revealed bilateral mixed hearing loss with bone conduction thresholds that were not age-appropriate (Fig. 1A). Other objective findings included marked thirst, and enlargement and induration of the bilateral submandibular and sublingual salivary glands. Nasal fibers showed no clear nasal polyps. Blood samples taken at the first visit were negative for both SS-A and SS-B antibodies, but a high serum IgG4 level of > 444 was observed. The gum test showed a marked decrease in saliva secretion (<1 mL/10 minutes). The patient also had subjective dry eye symptoms, and consulted the ophthalmology department of our hospital on the same day. When a Schirmer test was performed, the result was 5 mm/5 minutes, and the fluorescent dye test was strongly positive.

**Figure 2.**
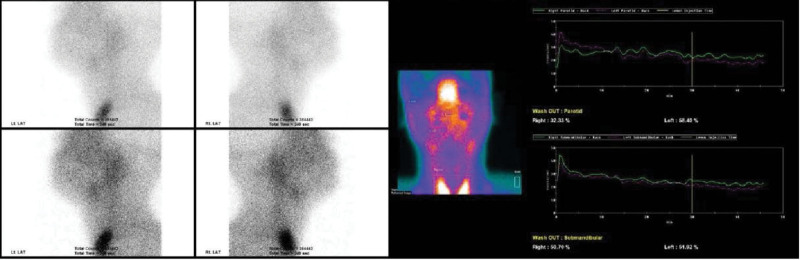
99mTc scintigraphy image. Accumulation in the bilateral parotid and submandibular glands is extremely low, and almost no accumulation is observed. In addition, almost no secretion by lemon juice stimulation is observed, suggesting severe functional decline. On the other hand, thyroid accumulation is normal.

At this point, the patient’s symptoms met the diagnostic criteria of IgG4-related Mikulicz’s disease (Table 1)^[1]^: “A item 1: Persistent (3 months or more), symmetrical enlargement of 2 or more pairs of lacrimal, parotid, and submandibular glands” and “A item 2: High serum IgG4 levels (135 mg/dL or higher).” In order to achieve a definite or probable diagnosis in the IgG4-RD follicle diagnostic criteria, pathological evaluation by tissue biopsy is required, as shown in item 3 of the comprehensive criteria (Table 2).^[2]^ However, simultaneously, the diagnostic criteria for SS related to ophthalmological symptoms (Table 3)^[3]^ were positive and marked hyposalivation was also observed. Based on the general principle of prioritizing the least invasive examinations for diagnosis, we decided to perform salivary gland scintigraphy prior to minor salivary gland biopsy and lacrimal gland biopsy. The results revealed “no function or severe functional decline” with almost no salivary secretion (Fig. 2).

**Table 1:** IgG4-related dacryoadenitis and sialoadenitis (IgG4-related Mikulicz’s disease).

1. At least 2 of the following: symmetrical enlargement of lacrimal, parotid, and submandibular glands (persisting for at least 3 mo)
2. High serum IgG4 level (≥135 mg/dL)
3. Infiltration of lymphocytes and IgG4 + plasma cells with characteristic fibrosis and sclerosis (IgG4/IgG > 0.5)
*Diagnosis is based on 1 and 2 or 1 and 3.

**Table 2:** Diagnostic criteria for IgG4-related disease comprehensive.

1. Diffuse or focal enlargement, tumor, nodes, or hypertrophic lesions in 1 or multiple organs
2. Hematologically high serum IgG4 levels (≥135 mg/dL)
3. The following 2 histopathological criteria: ① Significant infiltration and fibrosis of lymphocytes and plasma cells ② IgG4 + plasma cell infiltration: IgG4/IgG + cell ratio of ≥ 40% and > 10 IgG4 + plasma cells per high powered field (HPF) in biopsies
*Those who meet 1, 2, and 3: Definite diagnosis group
*Those who meet 1 and 3: Probable diagnosis group
*Those who meet 1 and 2: Possible diagnosis group
Diagnosis is possible based on diagnostic criteria for each organ even when definitive diagnosis based on clinical criteria is not possible.
In the case of probable or possible diagnosis based on comprehensive diagnostic criteria, which are used in combination with diagnostic criteria for organ-specific IgG4-related disease. The following has been published to date: “IgG4-related dacryoadenitis and sialoadenitis (IgG4-related Mikulicz’s disease) the diagnostic criteria,” “Diagnostic criteria for autoimmune pancreatitis,” “Diagnostic criteria for IgG4-related sclerosing cholangitis,” “Diagnostic criteria for IgG4-related nephropathy.”

**Table 3:** Revised Japanese criteria for Sjögren’s syndrome.

1. Any of the following positive findings on biopsy histopathology A) Lymphocytic infiltration of 1 focus or more per ¼ m^2^ in labial gland tissue B) More than 1 focus per ¼ m^2^ of lymphocyte infiltration in the lacrimal gland tissue
2. Any of the following positive findings on oral examination A) Abnormal findings of stage I (punctate opacities of 1 mm or less in diameter) or higher on sialography B) Decreased salivary secretion (10 mL or less in 10 min in gum test, or 2 g or less in 2 min in Saxon test) and findings of decreased function in salivary gland scintigraphy
3. Any of the following positive findings on ophthalmologic examination A) 5 mm/5 min or less in Schirmer test and positive in Rose Bengal test B) 5 mm/5 min or less in Schirmer test and positive fluorescent dye test
4. Any of the following positive findings in serum test A) Anti-SS-A antibody positive B) Anti-SS-B antibody positive
*If any 2 of the above items (1, 2, 3, and 4) are positive, Sjögren’s syndrome will be diagnosed.

As a result, the 2 diagnostic criteria for SS were met but conflicted with the exclusion item, so we were unable to diagnose IgG4-related Mikulicz’s disease. Thus the patient was diagnosed with SS, and treated with artificial saliva, Chinese herbal medicine, and carbocisteine for dry mouth symptoms, while low-dose macrolide administration was continued for persistent OME but treatment was not effective.

**Figure 3.**
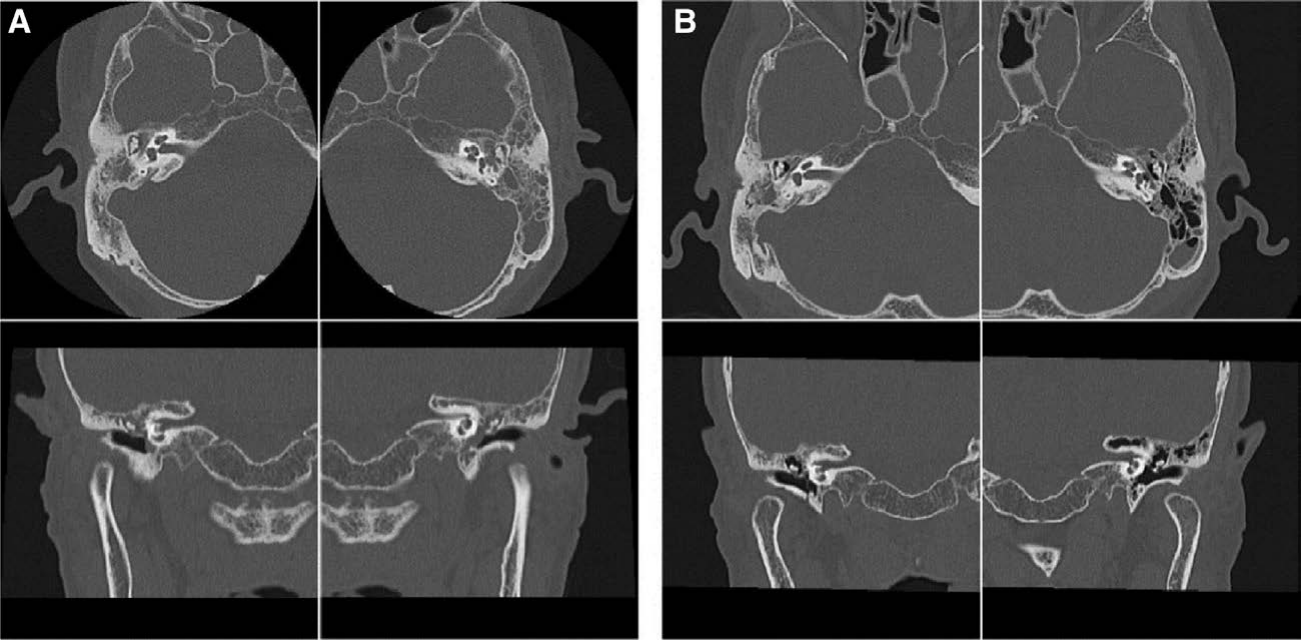
Changes in temporal bone CT image. (a): CT image of the temporal bone before starting treatment. Severe soft tissue opacities are observed bilaterally in the middle ear cavity and mastoid cells. (b): Temporal bone CT image during oral administration of 25 mg PSL/day, about 8 weeks after the start of oral steroid treatment. Bilateral otitis media with effusion improves, and the mastoid cells also puff up. CT = computed tomography, PSL = prednisolone.

A simple computed tomography scan of the neck, including the hearing apparatus, performed at the first visit to our department showed bilateral ethmoid and sphenoid sinus soft-tissue opacities, bilateral submandibular gland enlargement, and soft-tissue opacities in the bilateral middle ear cavities and mastoid cells were observed (Fig. 3A). The irreversible bilateral OME and mixed hearing loss were of greater concern than the dry mouth symptoms. Since the patient also had a history of bronchial asthma, we proposed temporary removal of the OME by myringotomy and a screening test for eosinophilic otitis media using an otorrhea eosinophil test; however, the patient did not consent to myringotomy and otorrhea sampling during the course of the study. No appearance or proliferation of nasal polyps was observed during the course of treatment. Although eosinophilic otitis media could not be ruled out, relatively high elevation bone conduction hearing thresholds had already been observed. Whether or not this mixed hearing loss (especially the increased bone conduction thresholds) is an otological sign of IgG4-RD, these clinical symptoms still interfere with daily life. Therefore, 3 months after the first visit, oral steroid treatment with prednisolone (PSL) was started after obtaining sufficient informed consent from the patient. There are no established standards for steroid administration for the otological signs of IgG4-RD. However, in IgG4-related Mikulicz’s disease, PSL generally starts at 0.6 mg/kg/day to induce remission and is gradually reduced over a long period of time and then maintained at a dosage of 5 to 7.5 mg/day^[4]^ starting at 30 mg/day (0.6 mg/kg/day for a patient weighing 50 kg in this case).

After the oral steroid treatment was initiated, early improvements in hearing and tympanic membrane findings were obtained, and the subjective symptoms of hearing loss were alleviated. The dose was reduced by 5 mg every 4 weeks, but after 16 weeks, when the dose decreased to 10 mg/day, the hearing loss worsened, so the dose was increased again to 15 mg/day which improved and stabilized the hearing (Fig. 1B, C, D). Since then, the patient has been on a maintenance PSL dose of 15 mg/day. At follow-up, 8 weeks after the start of treatment, computed tomography scans of the neck and ears were reexamined, and a significant improvement in soft tissue opacities was observed in the middle ear cavity, mastoid cellulite opacities, and sinus opacities (Fig. 3B).

Dry mouth symptoms did not improve, but induration enlargement of the bilateral submandibular and sublingual glands improved markedly. The serum IgG4 level also improved markedly from 444 mg/dL before the start of treatment to 183 mg/dL 8 weeks after the start of treatment. Additionally, various blood samples taken before the start of treatment were negative for p-antineutrophil cytoplasmic antibody (ANCA), c-ANCA, serum complement, antinuclear antibody, and sedimentation levels, and serum angiotensin converting enzyme, soluble IL-2R, and squamous cell carcinoma levels were not elevated. There were no signs of granulomatosis with eosinophilic polyangiitis (Churg-Strauss syndrome) or granulomatosis with polyangiitis (Wegener’s granulomatosis), sarcoidosis, Castleman’s disease, and malignant lymphoma or other carcinomas.

In this case, air and bone conduction thresholds on audiometric testing were used as an evaluation index of the therapeutic effect of treatment, and the PSL maintenance dose (approximately 15 mg/day) was quite high. The plan is to monitor the patient’s progress, paying attention to the possibility of adrenal insufficiency, steroid-induced DM complications, and osteoporosis.

## 3. Discussion and literature review

IgG4-RD is a disease characterized by high serum IgG4 levels, infiltration of IgG4 + plasma cells in 1 or multiple organs, and focal enlargement due to fibrosis, which result in masses, nodules, and hypertrophic lesions. Lacrimal sialadenitis, now known as Mikulicz’s disease, was first described in 1892 by the European surgeon Johann von Mikulicz-Radeki. The distinction between Mikulicz’s disease and SS, which presents with swollen salivary glands, has long been debated. However, in 1953, American pathologists Morgan and Castleman compared Mikulicz’s disease and SS and reported that they were histopathologically identical. For this reason, Mikulicz’s disease was considered a subtype of SS. However, in 2001, Hamano et al^[5]^ reported the relationship between autoimmune pancreatitis and IgG4. Yamamoto et al^[6]^ reported that Mikulicz’s disease presents with high serum IgG4 levels and IgG4 + plasma cell infiltration of the lacrimal and salivary gland tissues, while SS does not have these pathological findings, indicating the possibility that the 2 are different diseases. Since then, various systemic diseases, such as Küttner’s tumor, sclerosing cholangitis, retroperitoneal fibrosis, periarteritis, hypertrophic meningitis, interstitial pneumonia, and interstitial nephritis have been associated with IgG4. Consequently, it was proposed to collectively name these various organ lesions associated with IgG4 as “IgG4-related disease (IgG4-RD).” After such historical changes, in 2011, Umehara et al in Japan presented comprehensive diagnostic criteria for IgG4-RD (Table 2).^[2]^ Even if the diagnosis is partially definite or suspected according to the comprehensive diagnostic criteria (autoimmune pancreatitis, IgG4-related sclerosing cholangitis, IgG4-related Mikulicz’s disease, IgG-related renal lesions, etc.), it is possible to confirm the diagnosis using the diagnostic criteria for each organ. Although tissue biopsy has been suggested where possible, there are cases in which a diagnosis can be made without tissue biopsy. Among the diagnostic criteria for each organ, the criteria that we otorhinolaryngologists are most likely to encounter are those in suspected cases of IgG4-related Mikulicz’s disease (Table 1).^[1]^ Incidentally, serum IgG4 levels and histopathological findings may not meet these criteria even in cases clinically considered to be IgG4-related Mikulicz’s disease. On the contrary, as in this case, the serum IgG4 level may be high with bilateral and symmetrical enlargement of 2 or more organs, such as the lacrimal gland, parotid gland, and submandibular gland. Even when IgG4-associated Mikulicz’s disease is strongly suspected, if Schirmer/fluorescent dye test results, gum test results, and lacrimal tissue biopsy/minor salivary gland biopsy results meet the criteria for diagnosis of SS, IgG4-related Mikulicz’s disease may not be diagnosed due to exclusion criteria. In cases such as the present case, it is necessary to consider the diagnostic criteria and differentiation of IgG4-RD (especially IgG4-related Mikulicz’s disease) and SS. This is based on the premise that IgG4-related Mikulicz’s disease and SS are separate pathologies. However, apart from high serum IgG4 levels, and despite the symptoms similar to those of SS such as dry mouth and hypolacrimation, IgG4-related Mikulicz’s disease is characterized by a good response to treatment with steroids and good recovery of glandular function in some cases. However, in this case, the submandibular gland was significantly reduced, induration disappeared, and air and bone conduction hearing thresholds improved markedly, but dry mouth and salivary secretion did not improve. This difference in symptom improvement (although the enlargement of submandibular and sublingual glands significantly decreased and the induration disappeared, there was no improvement in salivary secretion) also made us consider the differences and differentiation between IgG4-related Mikulicz’s disease and SS.

The IgG4-RD comprehensive criteria states that “tissue biopsies should be performed whenever possible.” Therefore, we believe that biopsy of the submandibular gland, paranasal sinus mucosa, and middle ear mucosa may have revealed IgG4 + plasma cell infiltration in this case. However, if no nasal polyps that can be biopsied are found in the nasal cavity, or if consent cannot be obtained for otorrhea eosinophil testing by myringotomy, then obtaining consent for tissue biopsy of the submandibular gland, sublingual gland, and parotid gland tissue is considered even more difficult as a certain degree of invasiveness is required.

Several cases have been reported in which a definitive diagnosis of IgG4-RD was triggered by an otorhinolaryngological disease; Takagi et al ^[7]^ reported 5 cases of IgG4-RD with otitis media or hearing loss similar to this case (2 cases of otitis media, 2 cases of eosinophilic otitis media, and 1 case of sensorineural hearing loss). Among these cases, although the age of onset was middle-age and older, there was no clear difference in terms of sex, and serum IgG4 levels were high in all cases. Immunohistological examination of the lesions indicated that 4 of 5 cases had elevated IgG4/IgG levels (>40%). Incidentally, at our institution, we experienced a rare case wherein IgG4 + plasma cell infiltration was localized to the ossicles despite no increase in serum IgG4 level.^[8]^ In this case, the patient refused tissue sampling and consent was thus not obtained for otorrhea or middle ear mucosa sampling. Therefore, it was not possible to determine whether the condition of the middle ear cavity and mastoid cells was due to otological signs of IgG4-RD, eosinophilic otitis media, or a combination of both. Additionally, since the definitive diagnosis of SS was made by relatively minimally invasive examinations prior to biopsy of the submandibular and sublingual glands, it was difficult to actively perform tissue biopsy from the standpoint of defining the diagnosis. Furthermore, systemic steroid administration is considered a basic and effective treatment for symptoms and lesions in the otological region, regardless of whether the treatment is due to otological signs of IgG4-RD, eosinophilic otitis media, or a combination of both. Tissue biopsy remains important in IgG4-RD, SS, and eosinophilic otitis media, and it is highly desirable that tissue biopsy is performed for detailed examination of the pathology. However, in the comprehensive diagnostic criteria for IgG4-RD, a definitive diagnosis can be made when items 1 + 2 + 3 are met, including tissue biopsy, but even if the comprehensive diagnostic criteria are not completely met, satisfying organ-specific diagnostic criteria may enable diagnosis without necessarily requiring a tissue biopsy. In addition, in our country, SS is often diagnosed without tissue biopsy in certain cases using the revised Japanese criteria cases of SS that can be diagnosed without a tissue biopsy for Sjögren’s syndrome. Although this point of discussion remains uncertain, this case presents an opportunity for us to consider the following 2 points: If IgG4-RD (especially IgG4-related Mikulicz’s disease in the field of otorhinolaryngology) and SS have different pathologies, it may be necessary to exclude cases diagnosed with SS based on the exclusion criteria; In contrast, tissue biopsy (lacrimal glands, parotid and submandibular glands, lower labial minor salivary glands) might be necessary to diagnose SS (by making histological diagnosis a mandatory item, the histopathological evaluation can be reliably performed regardless of the results). However, we must always remember that tissue biopsies (specifically lacrimal gland biopsy, parotid gland biopsy, submandibular gland biopsy, lower lip minor salivary gland biopsy, and middle ear mucosa biopsy), and even nasal polyp biopsy and otorrhea by tympanostomy have aspects that impose a burden on the patient to some degree. Admittedly, the diagnostic criteria of the American College of Rheumatology (EULAR)^[9]^ differs from the Revised Japanese criteria for Sjögren’s syndrome, which allows for a definitive diagnosis to be made without a histological diagnosis (Table 3), used in this case. In the future, it may be necessary for Japan to follow the standards of the American College of Rheumatology, which are more stringent.

Despite the difficulty in managing this case, the diagnostic definition of IgG4-RD (especially IgG4-associated Mikulicz’s disease in the field of otorhinolaryngology) and SS should be further elucidated.

## 4. Conclusion

Clinically, although IgG4-related Mikulicz’s disease may be strongly suspected, it is necessary to distinguish it from SS based on its diagnostic definition. Careful interpretation of the diagnostic criteria is essential. Tissue biopsy may be essential in the diagnostic criteria for both IgG4-RD (including IgG4-related Mikulicz’s disease) and SS. More cases on otologic manifestations in IgG4-RD should be accumulated.

## Acknowledgments

We would like to thank Editage (www.editage.com) for English language editing.

## Author contributions

**Investigation:** Koichiro Oyake, Shuhei Uruma, Sawa Kamimura, Yojiro Kawamura.

**Methodology:** Koichiro Oyake, Shuhei Uruma, Sawa Kamimura, Yojiro Kawamura.

**Project administration:** Sei Kobayashi.

**Supervision:** Taketoshi Nogaki, Yoichi Ikenoya.

**Validation:** Taketoshi Nogaki, Yoichi Ikenoya.

**Visualization:** Naomi Imaizumi, Yukiko Inoue.

**Writing – original draft:** Yurie Otani, Tomotaka Shimura.

**Writing – review & editing:** Sei Kobayashi.
